# Author Correction: A nucleolar stress gene signature enables quantitative scoring across multi-omics contexts

**DOI:** 10.1038/s42003-026-10858-w

**Published:** 2026-09-03

**Authors:** Jianxiong Chen, Shuai Xiao, Zhe Hao, Huimeng Xu, Xuemei Xu, Jun Zhou

**Affiliations:** 1 Department of Pathology, The Tenth Affiliated Hospital, Southern Medical University (Dongguan People’s Hospital), Dongguan, China; 2 Dongguan Clinical Pathology Diagnosis Center, Dongguan, China; 3 Dongguan Key Laboratory of Clinical Pathology, Dongguan, China; 4 Department of Pathology, Nanfang Hospital, School of Basic Medical Sciences, Southern Medical University, Guangzhou, China; 5 Guangdong Province Key Laboratory of Molecular Tumor Pathology, Guangzhou, China

**Keywords:** Nucleolus, Mechanisms of disease

Correction to: *Communications Biology* https://doi.org/10.1038/s42003-026-10528-x, published online 29 June 2026

In the version of the article initially published, Jianxiong Chen was missing an affiliation (Department of Pathology, Nanfang Hospital, School of Basic Medical Sciences, Southern Medical University, Guangzhou, China) which has now been added. In the “Differential ribosome biogenesis and nucleolar stress at single-cell resolution” section, the sentence “We analyzed 371,223 tumors and matched adjacent normal cells from 62 CRC patients” should have read “We analyzed 371,223 single cells derived from tumor and matched adjacent normal tissues from 62 patients with CRC”. In the Fig. 5e legend, “Violin plots with overlaid box plots showing RiboSis (left) and NuS (right) across annotated tissue regions” should have read “Violin plots with overlaid box plots showing NuS (left) and RiboSis (right) across annotated tissue regions”. These corrections have been made to the HTML and PDF versions of the article.

