# Supplementary material for: A nucleolar stress gene signature enables quantitative scoring across multi-omics contexts
Source: Commun Biol. 2026 Jun 29;9:981. doi: 10.1038/s42003-026-10528-x (PMC13379586; doi:10.1038/s42003-026-10528-x)
Supplement: Supplementary file 2 — Supplementary Information [file 42003_2026_10528_MOESM2_ESM.pdf]

Supplementary Table 1.

Upregulated genes associated with nucleolar stress

Gene Symbol	Other Name(s)	Reference(s)
AATF		32587828
AKT1	AKT	24525337
ATF3		24098051
ATM		24675884, 37066425, 33146634, 27829214
ATR		40530693, 37066425, 33146634, 23447702, 25916852
BAX		36632465, 37645431
BBC3		40343270
CCDC137		40530693
CDKN1A	p21	40639572, 39705402, 31061518 etc, wildy reported
CDKN1B	P27KIP1	28981686
CDKN2A	p19ARF, p14ARF	28820908, 39528457, 25691462, 14636574, 22467867
CHEK1	CHK1	23447702
CNOT2		36569330
CORO2B	coronin 2B	38937439
CTNNB1	Beta-Catenin	33221336
E2F1		24675884
EEF2	eEF2	25332393
EEF2K	eEF2K	25332393
EMG1		39333759
FCN3		36632465
GADD45A		30177839
HIF1A		19680224, 17102617
HNRNPK		40338663
MAP3K5	ASK1	21383696
MAP3K8	TPL2	24998852
MAPK14	p38	21383696, 27445333
MDM2		22777350, 38859834
MDM4	MDMX	22777350

NEAT1		28288210
NCL		16751805
NOP53	GLTSCR2	22522597
NPM1	NPM, B23	15144954
KGD4	MRPS36	17131359
MYBBP1A		21297583
MYC	c-Myc	30247545, 21383696, 21383696
MYCN	N-Myc	34262099
NEDD8		22081073
PAK1IP1		21097889
PIM1		30247545
PML		15195100
PRKAA1	pAMPK	39521100
PRDM1		33972671
RGS6		38409136
RPL11		15152193, 12842086, 14612427, 16803902, 20194507, 21383696, 21807902, 21903592, 22262176, 22467867, 24141778, 26489471, 27975169, 30024791, 32198344, 22081073. 22588717
RPL22		29207594, 30874462, 39116201, 39146182
RPL23		31144295, 20554519, 29991511, 32236588, 15314174, 15314173, 22391559, 38981482
RPL26		20542919, 18951086
RPL3		25473889, 21705779, 23255119, 26636733, 32290083, 32244996, 28085118, 27835895, 27385096
RPL4		26908445
RPL5		22391559, 24141778, 32198344, 20554519, 35902361, 7935455, 22588717, 27265389
RPL6		24174547
RPS14		30706966
RPS2		31928715, 32370049
RPS25		22777350
RPS27		21170087

RPS27A	21561866
RPS27L	21170087
RPS7	30177839, 30247545, 19683495, 20554519
RPS9	36071402
SIRT7	32404984
SNORA13	38981482
SOCS3	23686487
SRSF1	23478443, 24807918
TOPBP1	25916852
TP53	40726293, 40639572, 39705402 etc, wildly reported
WNT4	32817586

References are listed with PubMed IDs (PMID).

Supplementary Table 2.

Downregulated genes associated with nucleolar stress		
**Gene Symbol**	**Other Name(s)**	**Reference(s)**
AGTPBP1		30905767
AKT1	AKT	23459592, 26993775
AKT1S1	PRAS40	24704832
BCL2		36632465, 37645431
BOP1		33510838, 38409361
BRIX1		39475053
CCDC137		40530693
CCND1	Cyclin D1	31659203
CDK4		30706966
CDK6		30706966
DHODH		32034120
DHX16		39333759
DHX33		36631557
E2F1		32535367, 33298840, 31659203, 35565259
EGR1		24098051
EIF2AK4	GCN2	37452637
EXOSC8		36348012
FANCA		33523834
FBXO7		31144295
FTO		40623539, 40213545
GNL3	NS, Nucleostemin	19033382, 21444791
GRK5		33507833
GRWD1		29991511, 28722511, 27856536
HEATR1		31190896, 37247644, 29143558
IGF1		20392698
IMPDH2		25347121, 39235732, 31371825
KMT5A	SETD8	39341827
LAS1L		34319761
LIN28A		34331666
MDM2		23807770，38285632, 22051195, 33298840
MDM4		17327702, 17110929, 38285632

MRPL44		40623539
MRPS16		40623539
MTREX	MTR4	36403484
MYC	c-myc	20194507, 24141778, 39341827, 38169774, 28288210, 21807902, 21807902, 37247644, 36569330, 31061518
NBN	NBS1	40067889
NCL		38409136
NDC80		39705402
NEDD8		25867069, 26993774
NFE2L2	NRF2	27445333
NIFK		25826659
NOD2		24098051
NOL12		30988155
NOL7		37246770
NOLC1	NOPP140	38062753, 23412656, 34201772, 30063880
NOP2		34319761
NOP53	PICT1	21804542, 16043506, 22320853, 36403484, 28722511, 27829214, 25464032, 24923447
NPM1		16855788, 22166220, 30814495, 21444791, 14636574, 33221336
NUF2		39705402
NUMA1		28981686
PELP1		34319761
PIM1		20639905, 26993775
POLR1A	RPA194	34572872, 31285544, 25344835
POLR1G	PAF49	37356716
PPAN		25759387, 30716409, 33221336, 36762786
PSMD9		34077860
PYGO2		23517060
RAP1GDS1	SmgGDS	28806394
RBM28		34953860
RIOK1		23459592, 29712692
RIOK2		23459592

RPL11		19129914, 27734913, 20221446
RPL13		35137207
RPL18		35137207
RPL23		24061479, 22588717
RPL27A		21674502, 32535367
RPL32		32516735
RPL37		20935493
RPL9		40623539, 40213545
RPS14		22391559
RPS15A		40623539, 40239541
RPS19		22391559, 23412928, 18515656, 27734913, 34757171
RPS2		33069875
RPS26		23728348
RPS27A		35073964
RPS27L		25144937
RPS6		19287375, 27734913
RPS6KA1	RSKS-1	29567958
RPS7		35871033, 19683495, 22588717
RPS8		35137207
RPS9		20221446
RRP12		26499779
RRP15		34343634, 28099941
RRP8		29567958
RRS1		34433556
SBDS		32198344, 36632465
SCD		36675264
SIRT1		34343634, 30623565
SPEN		37607001
SURF2		39333141
TAF1B		38169774, 37645431
TCOF1		15249688
TERF2	TRF2	29725012

TRIM24	TIF1A	24273493, 23764776, 37626880, 34572268, 36229381, 28360124, 29873780, 28334682
UBTF	UBF	30814495, 28806394, 26317157
USP47		32370049
UTP11		37087976
UTP14A		21078665
UTP18		20056613
WDR3		20392698
WDR5		30865883
WDR75		34611297
YBX1		36632465

References are listed with PubMed IDs (PMID).

Supplementary Table 3.

Proteins with altered subcellular localization in nucleolar stress response

Gene Symbol	Other Name(s)	Reference(s)
CDKN2A	p14ARF	39690184, 36230979, 16855788
E2F1		24675884
EIF6		36632465
GNL3	NS	36230979, 21464199, 39690184
HEY1		27129302
MAPT	Tau	35170061, 30064522
MYBBP1A		23583237, 21297583, 24710530, 24375404
NCL		21464199, 39690184
NOLC1		38617541
NPM1		24998852, 39690184, 38617541, 36825461, 36230979, 30301139
PARP1		38861757
PICT1		39690184
PPAN		25759387, 36762786
RAD9B		22399810
RBM28		34953860
REL1	p65	36230979, 30301139, 27445333, 37247644
RPL3		38617541
RPL5		25464032, 17327702, 17242401, 32516735, 17329973, 39690184
RPL6		24174547
RPL11		25464032, 17327702, 17242401, 14612427, 30024791, 32516735, 17329973, 39690184, 38409361
RPL23		17327702, 17242401M, 17329973, 19033382, 39690184
RPS7		39690184
RRN3		28334682
RSL1D1	CSIG	27811966
TERF2	TRF2	38062753
VHL		36230979

WDR12	PeBoW	27440937

References are listed with PubMed IDs (PMID).

Supplementary Figure 1.

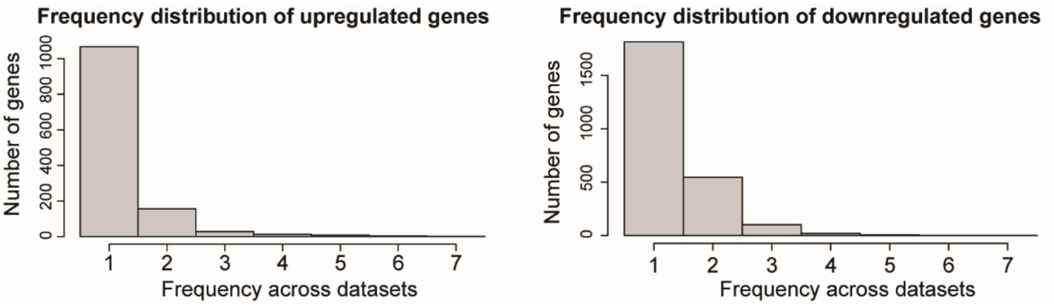

Supplementary Figure 1. Frequency of differentially expressed genes (DEGs) across nucleolar stress datasets. Histogram showing the distribution of DEG recurrence across 17 transcriptomic datasets, summarizing the occurrence frequencies of 1,277 upregulated and 2,489 downregulated genes.

Supplementary Figure 2.

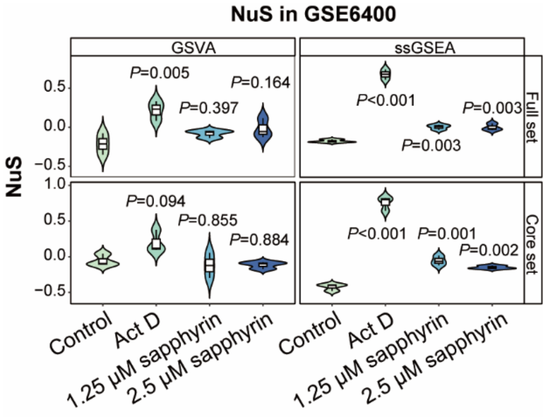

Supplementary Figure 2. ssGSEA- and GSVA-based quantification of nucleolar stress in A549 cells. NuS scores were calculated from the GSE6400 transcriptomic dataset using ssGSEA and GSVA. A549 cells were subjected to the indicated treatments (*n* = 3 in each group). Statistical significance was determined by one-way ANOVA with Dunnett's multiple-comparison test using the control group as the reference. Exact *P* values are indicated in the figure.

Supplementary Figure 3.

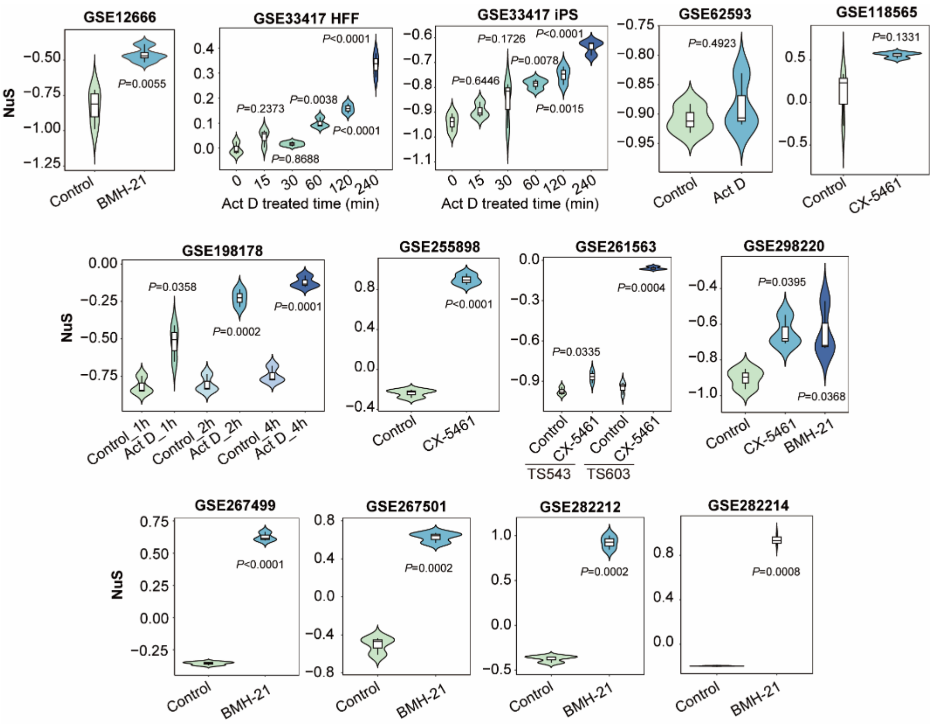

Supplementary Figure 3. Internal validation of the NuS across nucleolar stress datasets. NuS scores were calculated by ssGSEA with Full Set across 13 transcriptomic datasets. Sample sizes were n = 3 per group unless otherwise indicated (GSE12666 and GSE255898, n = 4 per group). Statistical analysis was performed using two-sided Welch's t-test for two-group comparisons, or one-way ANOVA with Dunnett's multiple-comparison test for multi-group comparisons, using untreated control samples as the reference. Exact P values are indicated in the figure.

Supplementary Figure 4.

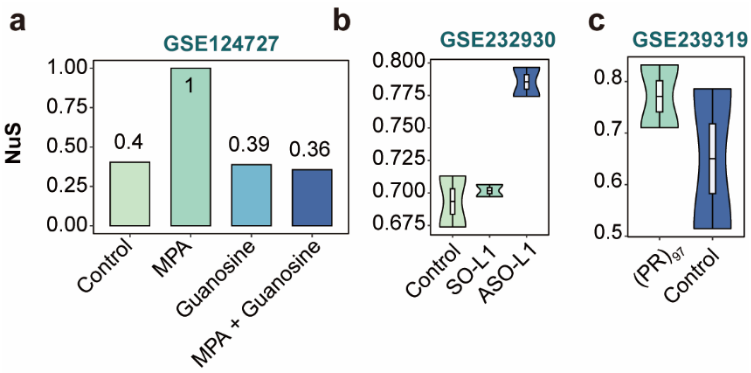

Supplementary Figure 4. NuS across independent public datasets. (a) GSE124727. NuS scores in cells treated with mycophenolic acid (MPA), guanosine, or MPA plus guanosine. (b) GSE232930. NuS scores in cells treated with a sense oligonucleotide (SO-L1) or a LINE1-targeting antisense oligonucleotide (ASO-L1). (c) GSE239319. NuS scores in samples with inducible expression of $(PR)^{97}$ and corresponding controls. Owing to the limited sample size (*n* = 1 in a; *n* = 2 per group in b and c), no statistical analysis was performed; the plots are shown for descriptive comparison only.

Supplementary Figure 5.

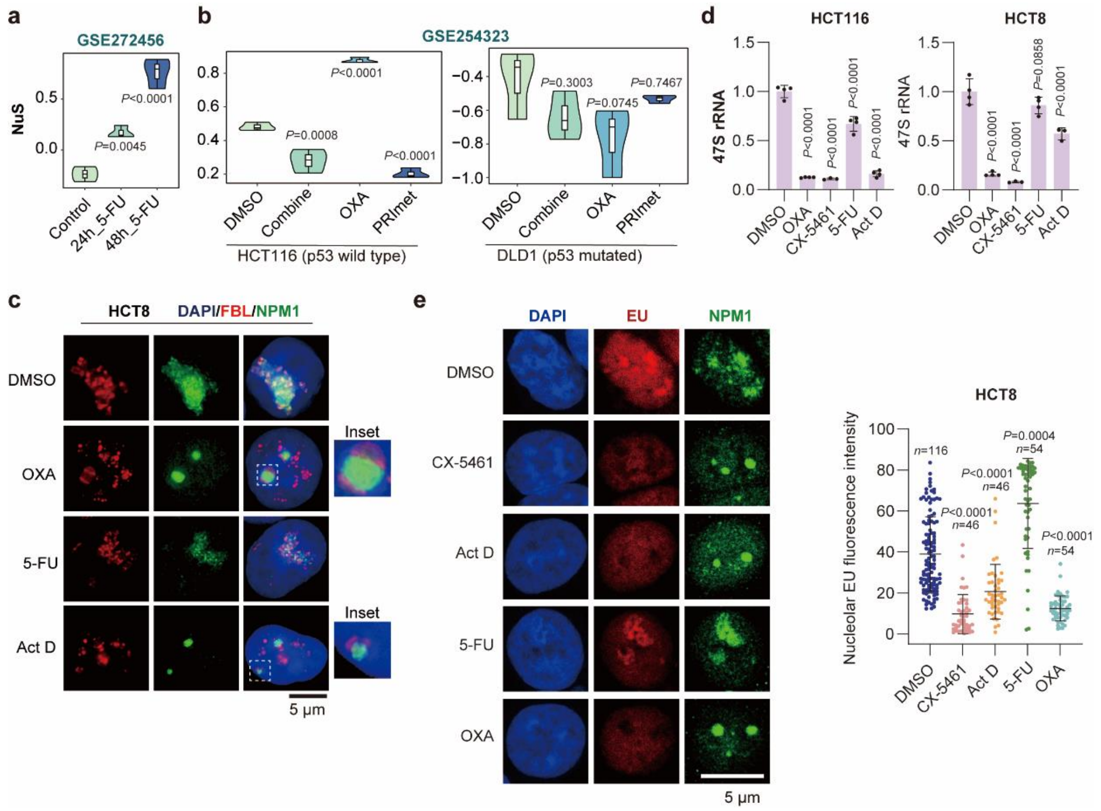

Supplementary Figure 5. Chemotherapeutic agents increase NuS and suppress rRNA transcription. (a) GSE272456. NuS scores in cells treated with 5-FU for 24 h or 48 h (n = 3 per group). Statistical analysis was performed using one-way ANOVA followed by Dunnett's multiple-comparison test, with the control group as the reference. (b) GSE254323. NuS scores in DLD1 (p53-mutant) and HCT116 (p53-wild-type) cells treated with oxaliplatin (OXA) or PRImet (n = 3 per group). Statistical analysis was performed using one-way ANOVA followed by Dunnett's multiple-comparison test, with DMSO as the reference group for each cell line. (c) SIM imaging of fibrillarin (FBL, red) and NPM1 (green) in HCT8 cells treated with DMSO, OXA, 5-FU or Act D. (d) qPCR analysis of 47S pre-rRNA levels in HCT116 and HCT8 cells treated with OXA, CX-5461, 5-FU or Act D. Statistical analysis was performed using one-way ANOVA followed by Dunnett's multiple-comparison test, with DMSO as the reference group. (e) 5-EU incorporation assay in HCT8 cells treated with the indicated agents. Representative images show 5-EU (red) and NPM1 (green). Right, quantification of nucleolar EU fluorescence intensity; each dot represents one nucleolus, with n indicated. Statistical significance was assessed using the Kruskal-Wallis test. Exact P values are indicated in the figure. Scale bar, 5 μm. Source data for d and e are provided in Supplementary Data 7.

Supplementary Figure 6.

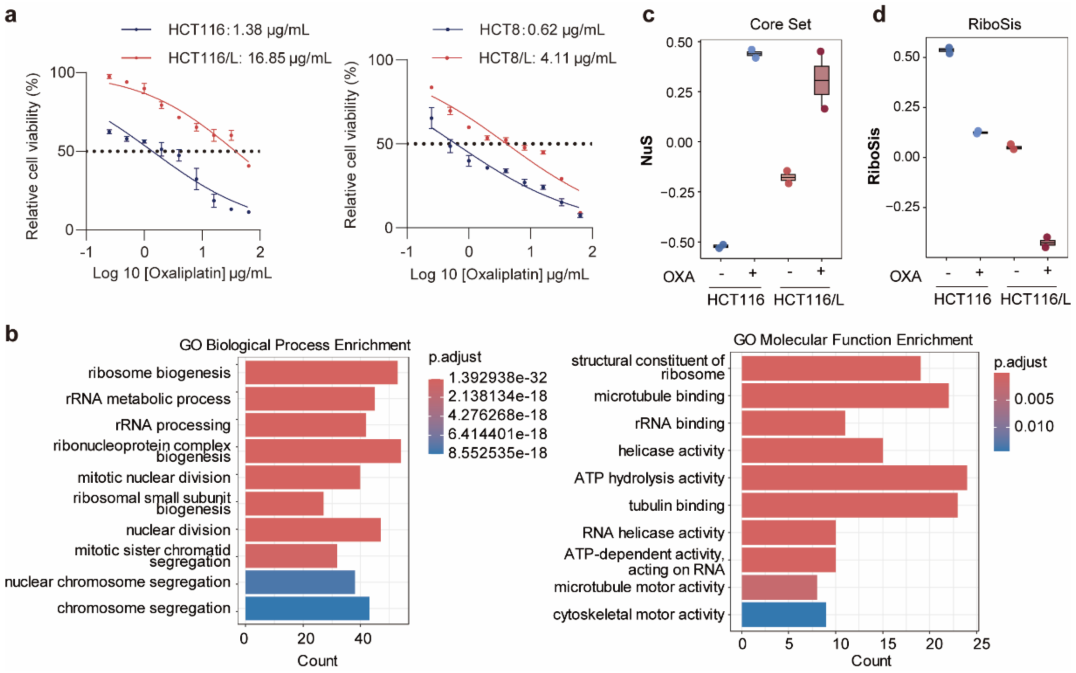

Supplementary Figure 6. Oxaliplatin sensitivity, pathway enrichment, and RiboSis changes in parental and resistant CRC cells. (a) Dose-response curves showing relative viability of HCT116, HCT116/L (oxaliplatin-resistant), HCT8, and HCT8/L cells following oxaliplatin treatment. (b) GO enrichment analysis of differentially expressed genes in oxaliplatin-treated HCT116 cells. (c-d) ssGSEA-based NuS (Core Set) and RiboSis scores in parental (HCT116) and resistant (HCT116/L) cells under control and oxaliplatin (OXA) treatment. Data are shown as boxplots (*n* = 2 biologically independent samples per group). Source data for a, c, and d are provided in Supplementary Data 7.

Supplementary Figure 7.

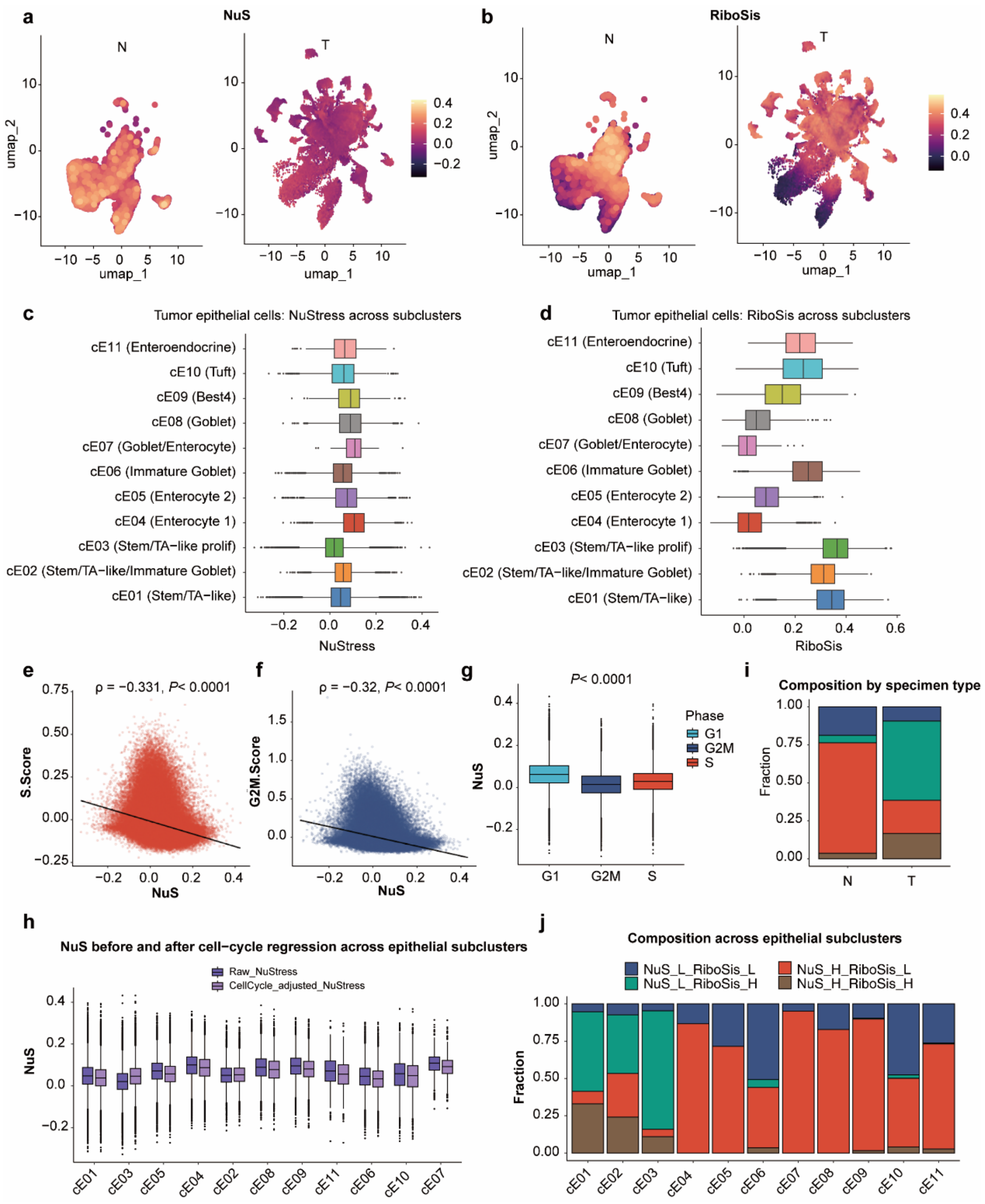

Supplementary Figure 7. Cell-cycle effects and epithelial heterogeneity of NuS and RiboSis at single-cell resolution. (a-d) Distribution of NuS (a, c) and RiboSis (b, d) across epithelial subpopulations. UMAPs show the spatial distribution of NuS (a) and RiboSis (b) in normal (N) and tumor (T) epithelial cells, and box plots summarize NuS (c) and RiboSis (d) across epithelial subclusters. (e, f) Spearman correlation analysis between NuS and cell-cycle scores. NuS showed a modest negative correlation with S-phase score (e) and G2/M score (f). Spearman's rank correlation coefficient (ρ) and corresponding *P* values are indicated. (g) Comparison of NuS across cell-cycle phases (G1, S, and G2/M). Statistical significance was assessed using the Kruskal-Wallis test. (h) Box plots showing NuS before and after regression

of cell-cycle effects across epithelial subpopulations. Adjusted NuS was obtained by regressing out S.Score and G2M.Score and re-centering the residuals, indicating that epithelial subcluster-specific variation in NuS remained evident after cell-cycle correction. (i, j) Composition of NuS/RiboSis-defined groups in normal and tumor epithelial cells (i) and across epithelial subpopulations (j).

Supplementary Figure 8.

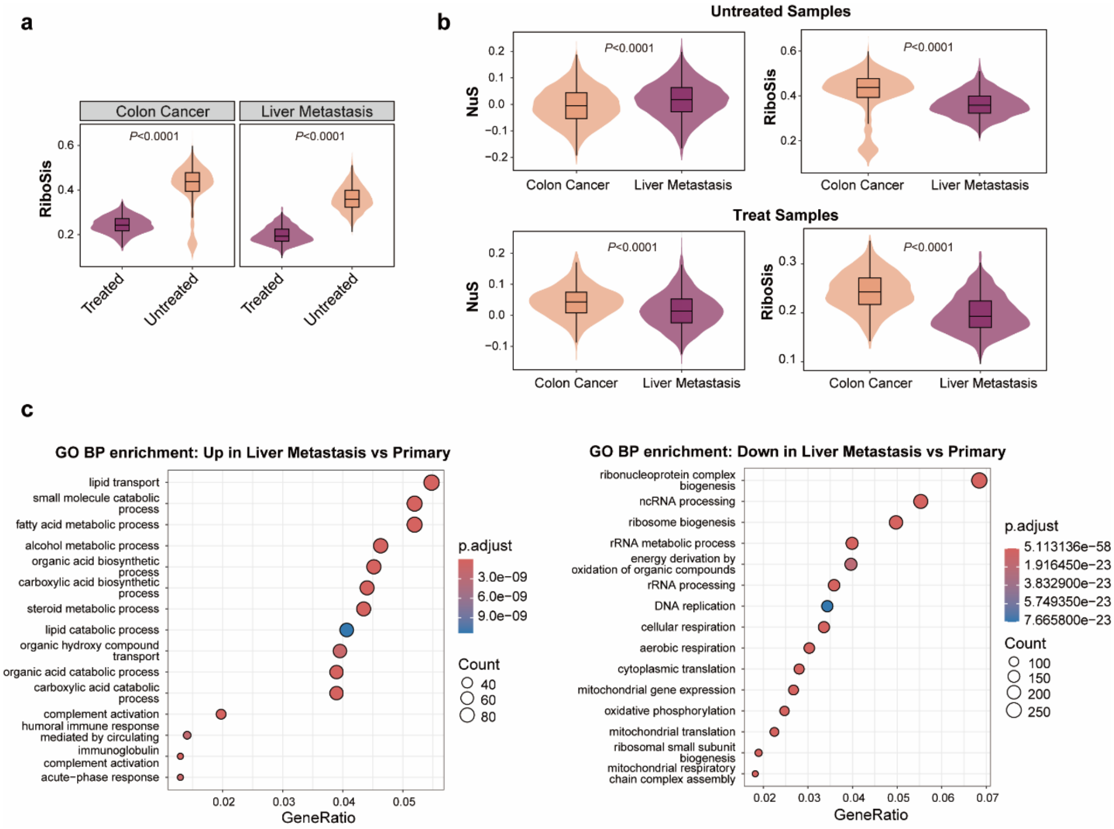

Supplementary Figure 8. NuS and RiboSis in primary tumors and liver metastases with and without neoadjuvant treatment. (a) Violin plots with overlaid box plots showing RiboSis in tumor regions from untreated and neoadjuvant-treated samples in primary tumors and matched liver metastases. Statistical significance was assessed using two-sided Wilcoxon rank-sum tests. (b) Violin plots with overlaid box plots comparing NuS between primary tumors and liver metastases within untreated and neoadjuvant-treated groups. Statistical significance was assessed using two-sided Wilcoxon rank-sum tests. (c) Gene Ontology (GO) biological process enrichment analysis of differentially expressed genes between liver metastases and primary tumors. Genes upregulated in liver metastases (left) and downregulated genes (right) are analyzed. Differential expression analysis was performed using Seurat FindMarkers with a two-sided Wilcoxon rank-sum test, and GO enrichment was conducted using clusterProfiler with Benjamini-Hochberg correction. Exact *P* values are indicated in the figure.

Supplementary Figure 9.

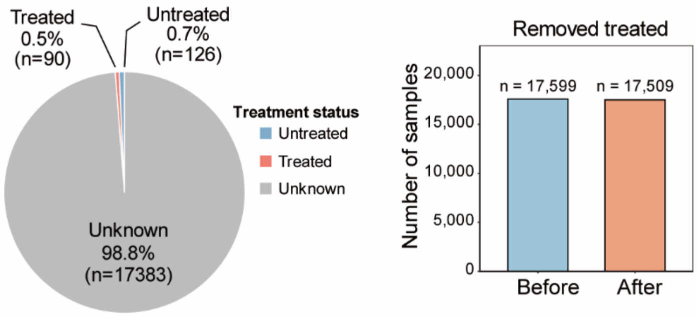

Supplementary Figure 9. Filtering of treatment-exposed samples. Proportion of untreated, treated, and treatment-unknown samples before filtering (left). Sample numbers before and after exclusion of treatment-exposed samples (right). Samples annotated as treated were removed to minimize confounding in downstream analyses.

Supplementary Figure 10.

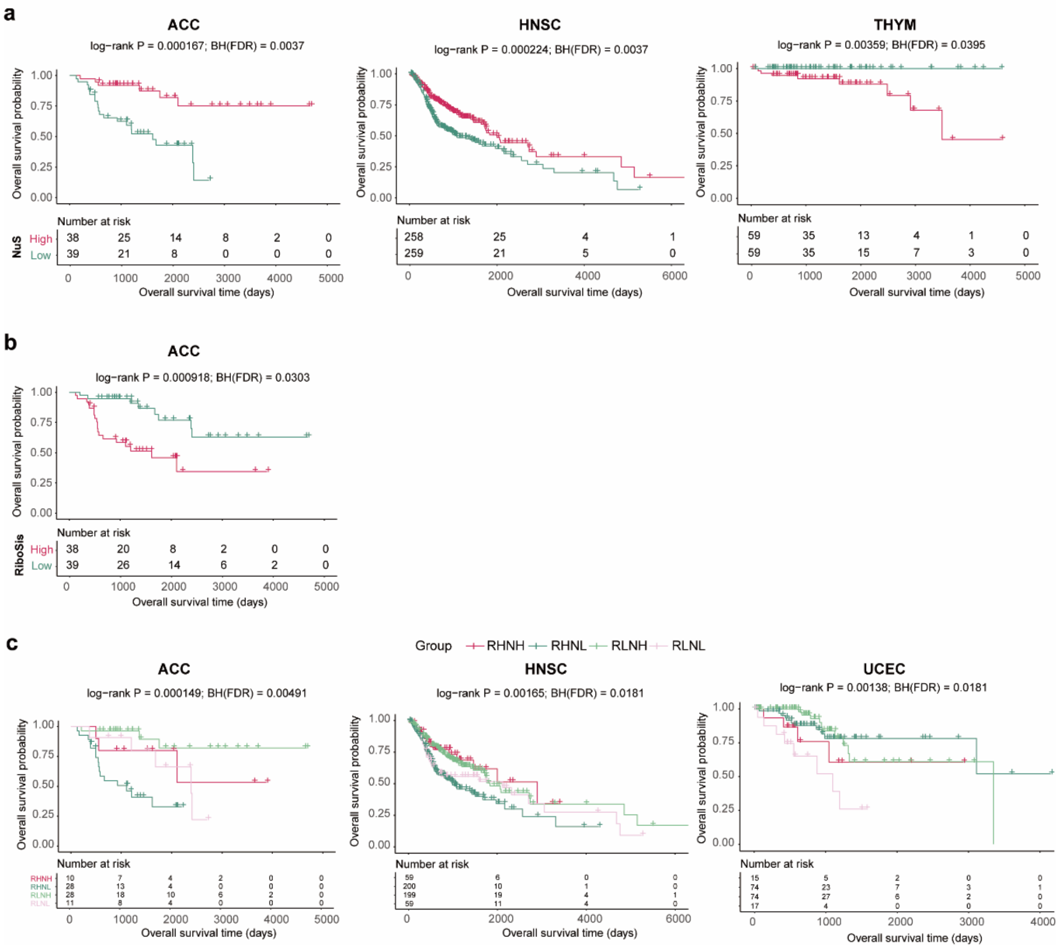

Supplementary Figure 10. Prognostic value of NuS and RiboSis in overall survival analysis. (a) Kaplan-Meier overall survival curves for patients stratified by NuS alone (high vs low, median cutoff). (b) Kaplan-Meier overall survival curves for patients stratified by RiboSis alone (high vs low, median cutoff). (c) Kaplan-Meier overall survival curves for patients stratified by the combined nucleolar functional states defined by NuS and RiboSis. Patients were classified into four groups using median cutoffs of NuS and RiboSis. Overall survival differences between groups were assessed using the log-rank test. Corresponding *P* values and Benjamini-Hochberg (BH)-adjusted *P* values are indicated in the plots. Hazard ratios (HRs) and 95% confidence intervals (CIs) were estimated using Cox proportional hazards models.

Supplementary Figure 11.

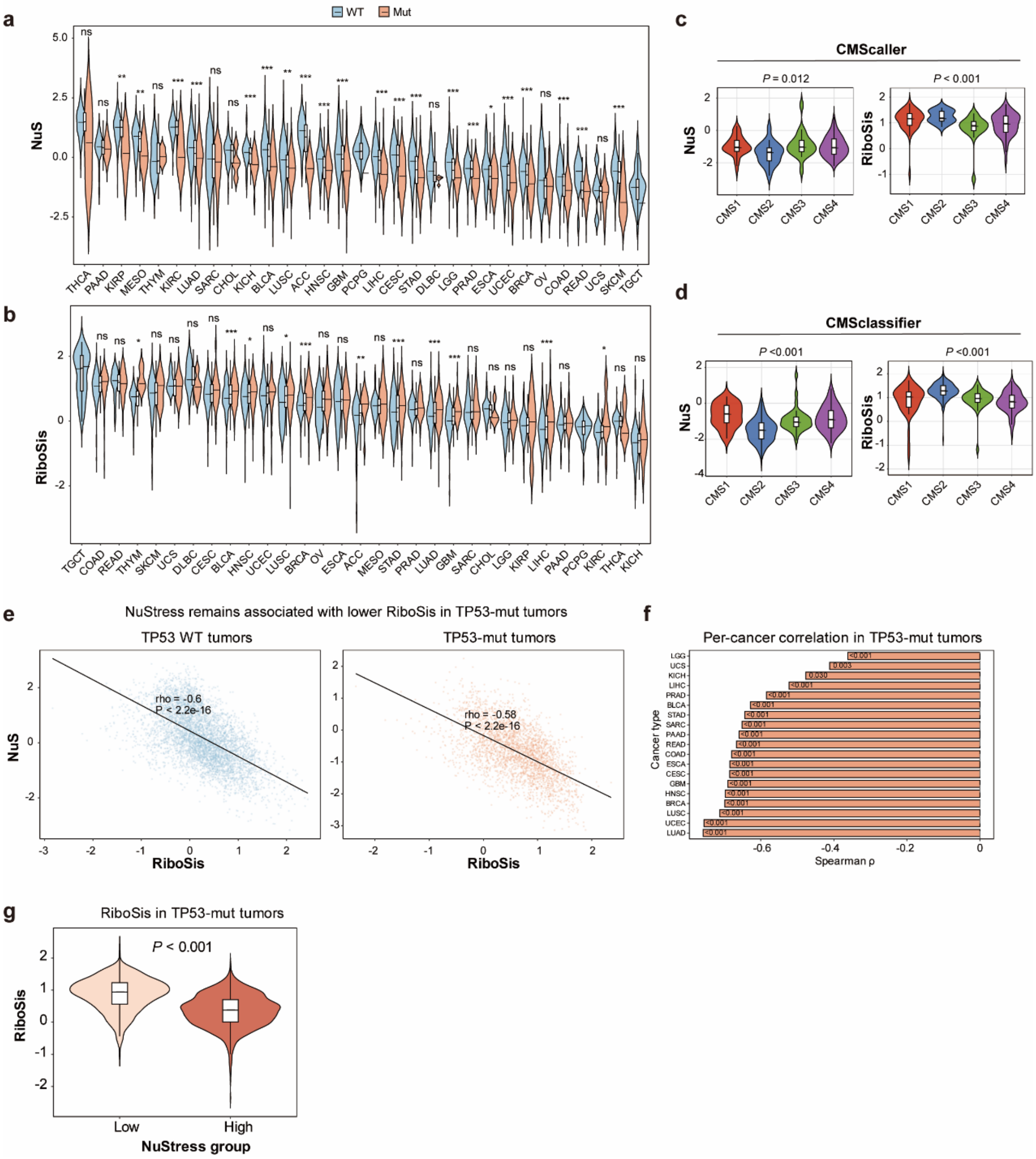

Supplementary Figure 11. Association of NuS and RiboSis with TP53 status and CRC subtypes. (a, b) Comparison of NuS (a) and RiboSis (b) between TP53 wild-type (WT) and TP53-mutant (Mut) tumor samples across cancer types. Statistical significance was assessed using a two-sided Wilcoxon rank-sum test. (c, d) Association of NuS and RiboSis with colorectal cancer (CRC) consensus molecular subtypes (CMS) defined using two classification methods, CMScaller (c) and CMSclassifier (d). NuS and RiboSis were compared across CMS groups. Statistical significance was evaluated using the Kruskal-Wallis test followed by post hoc multiple-comparison testing. (e) Correlation between NuS and RiboSis in TP53 wild-type and TP53-mutant tumor samples. Each dot represents one sample; Spearman correlation coefficients (ρ) and corresponding *P* values are shown. (f) Per-cancer correlation analysis between NuS and RiboSis in TP53-mutant tumors. Bars indicate Spearman correlation

coefficients for individual cancer types. (g) Comparison of RiboSis levels between NuS-defined groups (high versus low) in TP53-mutant tumors. Statistical significance was assessed using a two-sided Wilcoxon rank-sum test. ns, not significant; * $P < 0.05$; ** $P < 0.01$; *** $P < 0.001$. Exact *P* values are indicated in the Supplementary Data 8.

Supplementary Figure 12

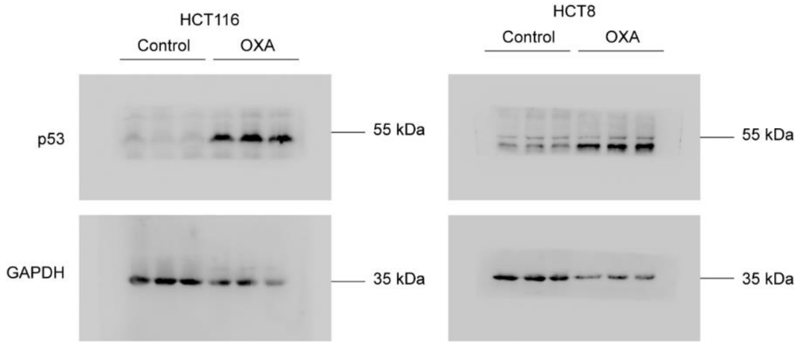

Supplementary Figure 12. Unprocessed images of Figure 3h.
