# Supplementary material for: A nucleolar stress gene signature enables quantitative scoring across multi-omics contexts
Source: Commun Biol. 2026 Jun 29;9:981. doi: 10.1038/s42003-026-10528-x (PMC13379586; doi:10.1038/s42003-026-10528-x)
Supplement: Supplementary file 3 — Description of Additional Supplementary Files [file 42003_2026_10528_MOESM3_ESM.pdf]

Description of Additional Supplementary files

Supplementary Data 1. Literature-curated nucleolar stress-responsive genes.

Supplementary Data 2. Overview of public GEO transcriptomic datasets, preprocessing characteristics, reanalysis methods, and differential expression results.

Supplementary Data 3. Full list of differentially expressed genes identified across all 17 datasets.

Supplementary Data 4. Dataset-derived nucleolar stress gene set after frequency filtering.

Supplementary Data 5. Full Set of nucleolar stress-associated genes.

Supplementary Data 6. Core Set of high-confidence nucleolar stress-associated genes.

Supplementary Data 7. Source data underlying all main figures and supplementary figures.

Supplementary Data 8. Exact p values for statistical analyses not reported directly in the figures.
