# Supplementary material for: A nucleolar stress gene signature enables quantitative scoring across multi-omics contexts
Source: Commun Biol. 2026 Jun 29;9:981. doi: 10.1038/s42003-026-10528-x (PMC13379586; doi:10.1038/s42003-026-10528-x)
Supplement: Supplementary file 1 — Transparent Peer Review file [file 42003_2026_10528_MOESM1_ESM.pdf]

Peer Review File

A Nucleolar Stress Gene Signature Enables Quantitative Scoring Across Multi-Omics Contexts

Corresponding Author: Professor Jun Zhou

This file contains all reviewer reports in order by version, followed by all author rebuttals in order by version.

Version 0:

Reviewer comments:

Reviewer #1

(Remarks to the Author)
The manuscript presents an intriguing and highly novel study on a nucleolar stress signature (NuS) in cancer, with a well-executed bioinformatics analysis. The authors are to be commended for their comprehensive approach to integrating multi-omics data and deriving a biologically meaningful signature linked to nucleolar stress and p53 signaling.
However, several major concerns require attention to enhance clarity, methodological rigor, reproducibility, and biological interpretability of the findings. Addressing these points would substantially strengthen the manuscript.

1. Differential expression analysis across heterogeneous microarray platforms
The authors applied a uniform differential expression analysis pipeline using limma across multiple microarray datasets, employing the common thresholds of $|log_2FC| > 1$ and adjusted $P < 0.05$. Although computationally efficient, this strategy risks introducing platform-specific biases unless preprocessing steps (background correction, normalization, batch effect correction) are carefully tailored to each microarray technology (e.g., Affymetrix vs. Illumina). For instance, RMA or GCRMA is standard for Affymetrix arrays, while quantile normalization or variance-stabilizing normalization (VSN) may be preferable for others. Moreover, integrating datasets from different platforms without robust batch correction (e.g., via ComBat or surrogate variable analysis [SVA]) can generate spurious differentially expressed genes (DEGs) driven by technical rather than biological variation.
To improve robustness and reproducibility of the DEG calls—and thus the derived NuS—the authors should explicitly describe in the revised Methods section:
the preprocessing steps applied to raw data from each platform;
whether platform-specific normalization methods were used.
These clarifications are essential for validating the nucleolar stress signature.

2. Relationship of the NuS signature to Consensus Molecular Subtypes (CMS) in colorectal cancer (CRC)
CRC is characterized by a well-established and widely adopted molecular classification system—the Consensus Molecular Subtypes (CMS)—derived from integrative gene expression analysis (Guinney et al., Nat Med. 2015;21:1350–1356). Given the central role of p53 signaling in the authors' nucleolar stress framework, it would be highly informative to evaluate how NuS scores relate to these biologically and clinically distinct subtypes (CMS1: MSI/immune-activated; CMS2: canonical/WNT-MYC; CMS3: metabolic; CMS4: mesenchymal/stromal/TGF-β). For example, does NuS preferentially enrich in or complement specific CMS groups?
The authors are strongly encouraged to apply a CMS classifier (e.g., the CMScaller R package, available at https://github.com/peterawe/CMScaller) to their bulk (or pseudobulk) CRC samples and interpret NuS scores in the context of CMS assignments. This analysis would provide important insights into the clinical and biological relevance of the signature.

3. Association between TP53 alterations and NuS scores
Since p53 pathway activation is a core theme of the study, a formal statistical assessment of the relationship between TP53 status (mutations, deletions, or loss) and elevated NuS scores is warranted. Appropriate methods include Fisher's exact test, chi-squared test, or logistic regression (potentially adjusting for covariates). Such an analysis would clarify whether NuS primarily captures p53-dependent nucleolar stress responses in vivo or reflects more general defects in ribosome biogenesis that are independent of p53 status.

4. Potential confounding by cell cycle heterogeneity
Ribosome biogenesis and rRNA processing genes are often cell cycle–regulated, with many downregulated during G0/G1 or mitosis. Consequently, NuS may partly reflect proliferation status or cell cycle phase distribution rather than specific nucleolar stress. This is particularly relevant in single-cell datasets, where cell cycle heterogeneity is prominent.
To address this, the authors should compute established cell cycle scores (e.g., using Seurat's CellCycleScoring function with canonical S-phase and G2/M-phase gene sets) and evaluate correlations between NuS and cell cycle phase across datasets. If a strong covariance with proliferation is observed, this should be explicitly acknowledged. Ideally, the signature could be refined (e.g., by regressing out cell cycle effects) to better isolate nucleolar stress-specific signals. This control experiment would greatly bolster the biological specificity of NuS.

5. Distinguishing tumor-intrinsic effects from tissue-of-origin or microenvironmental influences
To confirm that differences in NuS (and related ribosome biogenesis signatures, RiboSis) reflect tumor-intrinsic oncogenic processes rather than baseline tissue-specific or microenvironmental nucleolar states, inclusion of a negative control cohort is recommended. Healthy tissues from multiple organs (e.g., from GTEx) would serve as an appropriate reference to benchmark NuS levels in non-malignant contexts. Without such controls, it remains uncertain whether observed elevations are truly cancer-specific.

The study offers valuable insights into nucleolar stress in cancer, addressing these methodological and interpretative issues will markedly improve its scientific impact, rigor, and potential for clinical translation.

Reviewer #2

(Remarks to the Author)
The authors describe a score for measuring nucleolar stress (NuS), applicable to a wide variety of data types. They evaluate the score in the context of colorectal cancer using both single-cell RNA-seq and spatial transcriptomic and further evaluate NuS using TCGA and CMap datasets, comparing with results given by the ribosomal scoring method RiboSis.

Overall, I find the paper to be very well written, with robust analyses generated across numerous datasets and data types. I have the following specific comments, mainly around validation:

1. Lines 241 – 250 and Figures 4k-n. The authors stratify epithelial cell subsets where low-NuS cells show pathways related to ribosome biogenesis and rRNA metabolism that are downregulated. The claim is made that NuS is capturing functional variation not explained by Ribosome biogenesis alone. However, across the epithelial cell subsets, the RiboSis scores roughly seem to correspond to the NuS score. E.g. in the cE01 to cE03 subsets, both the RiboSis and NuS scores are elevated. Does the ribosome biogenesis etc. GO terms refer to the low RiboSis score here? What happens if the stratifications are performed based on RiboSis instead? What is the overlap of the cell stratifications, and do the GO terms look different?

2. Lines 269-278: the therapy was associated with an increase in the NuS score. Do the authors know if the specific therapy is expected to induce nucleolar stress? That would help with validation. Similarly, the authors comment based on the RiboSis and NuS scores that the metastatic tumours may adopt a less proliferative state. This should be something that the authors could test with differential gene expression and GO term analysis?

3. The authors evaluated the NuS score across drugs in CMap. In Potapova et al. eLife 2013 (PMID: 38099650), they scanned anti-cancer drugs for impact on nucleolar stress. As a validation, it would be good to compare the normality scores from Potapova et al. to the NuS scores, where there is overlap in the drugs tested.

4. The authors state in lines 336 – 337 that the recovery of Nutlin-3 is an internal validation, but I would like clarification here. In Goudarzi et al. it is stated “Nutlin-3 activates p53 by disruption of the p53-MDM2 protein-protein interaction and without inducing nucleolar stress”. Can the authors clarify where they get their understanding of Nutlin-3 as a canonical nucleolar stress inducer? In either case, the comparison with the Potapova et al. dataset would make for a more robust testing of the NuS score.

5. In figure legends, the authors should indicate what statistical test is being used, for example, in Figure 3g, Figure 4k-l, Figure 5f, Figure 6a, Supp Figure 8c. etc.

Reviewer #3

(Remarks to the Author)
I co-reviewed this manuscript with one of the reviewers who provided the listed reports. This is part of the Communications Biology initiative to facilitate training in peer review and to provide appropriate recognition for Early Career Researchers who co-review manuscripts.

Reviewer #4

(Remarks to the Author)
Chen el al. present a methodology and analytical framework to study nucleolar stress using a quantitative computational

metric (NuS), followed by hypothesis-driven experimental testing and biological validation. The authors curate knowledge across the literature and multiple published expression datasets to identify a set of genes associated with nucleolar stress, addressing the current lack of a well-defined gene signature or ontology for this process. Conceptually, NuS is related to the RiboSis metric developed by another group; however, whereas RiboSis is derived from a predefined set of known ribosome biogenesis genes, this study seeks to define a new nucleolar stress–associated gene set and subsequently formalize it into a quantitative score. From my perspective, a key innovation of this work lies in its use of computational analysis as a means for hypothesis generation and prioritization of downstream experimental validation.

My feedback:
1. Specificity of NuS to nucleolar stress
My main concern relates to the specificity of NuS for nucleolar stress, as opposed to reflecting more general cellular stress or damage responses. For example, in "Oxaliplatin induces nucleolar stress in colorectal cancer", NuS also increased following 5-FU treatment, despite the absence of clear nucleolar morphological changes. This raises the question of whether NuS selectively captures nucleolar stress or instead reflects broader transcriptional programs associated with cytotoxicity or stress responses.
The authors may wish to clarify this distinction, for example by (i) benchmarking NuS against established stress signatures (e.g. DNA damage, ER stress, or general p53 activation), or (ii) explicitly testing conditions known to induce non-nucleolar stress.
2. p5 line 119 "18 upregulated and 33 downregulated" are not listed in Supplementary Data 2.
3. p5 lines 123-126 I suggest restricting the DepMap essentiality analysis to the upregulated genes. Downregulation of a gene does not necessarily imply functional dependency or essentiality.
4. Relationship between NuS and RiboSis
While the manuscript appropriately positions NuS as complementary to RiboSis, it would benefit from a more explicit discussion of overlap and divergence between the two metrics. For example, quantifying gene-level overlap or correlation across datasets would help clarify how these scores capture distinct versus shared aspects of nucleolar and ribosome-related biology.
5. Causality versus association
The manuscript would benefit from clearer language regarding the relationship between NuS and nucleolar stress. In particular, it would be helpful to clarify whether changes in NuS are intended to precede, coincide with, or follow experimental indicators of nucleolar stress, and to explicitly state that NuS represents an associative metric rather than a causal measure.

Version 1:

Reviewer comments:

Reviewer #1

(Remarks to the Author)
After reviewing the authors' detailed response to my earlier remarks, I would like to express my satisfaction with the revisions implemented. The authors have addressed the raised concerns in a constructive and comprehensive manner, resulting in a noticeably improved manuscript that demonstrates greater rigor, analytical precision, and well-supported interpretations of the results and their broader implications.
The only remaining minor point pertains to Figure 5. To enhance visual consistency and enable more straightforward interpretation, I recommend aligning the color palettes assigned to the individual cell clusters. In particular, the palettes utilized in subpanels (b) and (e) should be standardized.
This minor adjustment would further elevate the clarity and professionalism of the presentation without altering the scientific content.

Reviewer #2

(Remarks to the Author)
I thank the authors for addressing my comments.

Reviewer #3

(Remarks to the Author)
I co-reviewed this manuscript with one of the reviewers who provided the listed reports. This is part of the Communications Biology initiative to facilitate training in peer review and to provide appropriate recognition for Early Career Researchers who co-review manuscripts.

Reviewer #4

(Remarks to the Author)
The authors have addressed all my comments.

Open Access This Peer Review File is licensed under a Creative Commons Attribution 4.0 International License, which permits use, sharing, adaptation, distribution and reproduction in any medium or format, as long as you give appropriate credit to the original author(s) and the source, provide a link to the Creative Commons license, and indicate if changes were made.
In cases where reviewers are anonymous, credit should be given to 'Anonymous Referee' and the source.
The images or other third party material in this Peer Review File are included in the article's Creative Commons license, unless indicated otherwise in a credit line to the material. If material is not included in the article's Creative Commons license and your intended use is not permitted by statutory regulation or exceeds the permitted use, you will need to obtain permission directly from the copyright holder.
To view a copy of this license, visit https://creativecommons.org/licenses/by/4.0/

RESPONSE TO REVIEWERS' COMMENTS

***Note to all reviewers:*:**

We sincerely thank the reviewers for their careful assessment of our manuscript and for their constructive and insightful comments. Their feedback has been invaluable in helping us improve the quality and clarity of the paper. Below, we provide a detailed point-by-point responses to all comments raised. To facilitate evaluation, we summarize the key data and revisions in this response letter and indicate the corresponding revised figures and text in the manuscript where appropriate. All changes in the revised manuscript are indicated in blue.

Reviewers' comments:

========================

Reviewer #1 (Remarks to the Author):

The manuscript presents an intriguing and highly novel study on a nucleolar stress signature (NuS) in cancer, with a well-executed bioinformatics analysis. The authors are to be commended for their comprehensive approach to integrating multi-omics data and deriving a biologically meaningful signature linked to nucleolar stress and p53 signaling.
However, several major concerns require attention to enhance clarity, methodological rigor, reproducibility, and biological interpretability of the findings. Addressing these points would substantially strengthen the manuscript.

1. Differential expression analysis across heterogeneous microarray platforms

The authors applied a uniform differential expression analysis pipeline using limma across multiple microarray datasets, employing the common thresholds of $|log_2FC| > 1$ and adjusted $P < 0.05$. Although computationally efficient, this strategy risks introducing platform-specific biases unless preprocessing steps (background correction, normalization, batch effect correction) are carefully tailored to each microarray technology (e.g., Affymetrix vs. Illumina). For instance, RMA or GCRMA is standard for Affymetrix arrays, while quantile normalization or variance-stabilizing normalization (VSN) may be preferable for others. Moreover, integrating datasets from different platforms without robust batch correction (e.g., via ComBat or surrogate variable analysis [SVA]) can generate spurious differentially expressed genes (DEGs) driven by technical rather than biological variation. To improve robustness and reproducibility of the DEG calls—and thus the derived NuS—the authors should explicitly describe in the revised Methods section: the preprocessing steps applied to raw data from each platform; whether platform-specific normalization methods were used. These clarifications are essential for

validating the nucleolar stress signature.

Our response:

We thank the reviewer for this important comment. In the revised manuscript, we have clarified our analytical strategy in both the Methods section and Supplementary Data 2.

First, we emphasize that our study did not perform differential expression analysis by merging expression matrices across platforms into a single integrated dataset. Instead, each dataset was analyzed independently using a workflow appropriate to its data type. Candidate nucleolar stress-associated genes were subsequently defined based on cross-dataset recurrence with consistent directionality, rather than direct cross-platform integration. This design minimizes the risk that platform-specific technical variation drives gene selection.

Accordingly, we did not apply a single uniform preprocessing pipeline across all datasets. For each dataset, preprocessing and transformation were handled according to the format of the deposited data and the procedures described in the original studies. Specifically, for microarray datasets, we used processed GEO matrices, mapped probe identifiers using platform-specific annotation resources, and collapsed duplicated gene symbols by retaining the feature with the highest median expression. For RNA-seq datasets, processed TPM or FPKM matrices were used, with appropriate filtering and $\log_2(x + 1)$ transformation applied when necessary. For the Agilent time-course dataset GSE62963, the deposited matrix was already normalized to the pretreatment baseline and was therefore analyzed as a baseline-normalized $\log_2$ fold-change matrix rather than a conventional expression matrix.

Because datasets were not combined at the raw expression level, batch correction methods such as ComBat or SVA were not applicable. These approaches are primarily designed for harmonizing jointly analyzed datasets, whereas our framework relies on within-dataset DEG identification followed by frequency-based integration across studies, thereby reducing susceptibility to cross-platform technical artifacts.

To improve transparency and reproducibility, we have revised the Methods section to explicitly describe: (i) the preprocessing status of each dataset as provided in GEO; (ii) the transformation strategies applied to different data types; (iii) the DEG analysis framework used for each dataset; and (iv) the cross-dataset recurrence-based strategy used to define robust candidate genes. In addition, we have summarized key dataset-level information in Supplementary Data 2, including platform, matrix type, preprocessing status, statistical methods, and DEG counts.

2. Relationship of the NuS signature to Consensus Molecular Subtypes (CMS) in colorectal cancer (CRC)

CRC is characterized by a well-established and widely adopted molecular classification system—the Consensus Molecular Subtypes (CMS)—derived from integrative gene expression analysis (Guinney et al., Nat Med. 2015;21:1350–1356). Given the central role of p53 signaling in the authors' nucleolar stress framework, it would be highly informative to evaluate how NuS scores relate to these biologically and clinically distinct subtypes (CMS1: MSI/immune-activated; CMS2: canonical/WNT-MYC; CMS3: metabolic; CMS4: mesenchymal/stromal/TGF-β). For example, does NuS preferentially enrich in or complement specific CMS groups? The authors are strongly encouraged to apply a CMS classifier (e.g., the CMScaller R package, available at https://github.com/peterawe/CMScaller) to their bulk (or pseudobulk) CRC samples and interpret NuS scores in the context of CMS assignments. This analysis would provide important insights into the clinical and biological relevance of the signature.

Our response:

We thank the reviewer for this insightful suggestion. We agree that evaluating the relationship between NuS and Consensus Molecular Subtypes (CMS) provides important biological and clinical context for interpreting nucleolar stress in CRC.

In the revised manuscript, we have incorporated CMS-based analyses using two independent classification frameworks, including the CMScaller and CMSclassifier algorithms, to ensure robustness and consistency of subtype assignment. As shown in Supplementary Figure 11c-d, NuS and RiboSis exhibit distinct and subtype-specific distributions across CMS groups.

Specifically, we observed that NuS is lowest in CMS2 tumors, whereas RiboSis is highest in this subtype, consistent with the well-established enrichment of MYC signaling and ribosome biogenesis programs in CMS2. In contrast, other CMS subtypes display relatively higher NuS levels, reflecting increased activation of stress-associated and microenvironment-related programs. These patterns were consistently observed across both classification methods, supporting the robustness of the findings.

We have added a dedicated description of CMS classification and its integration with NuS analysis in the Methods section, and expanded the corresponding Results section to highlight subtype-specific patterns of NuS and their biological interpretation.

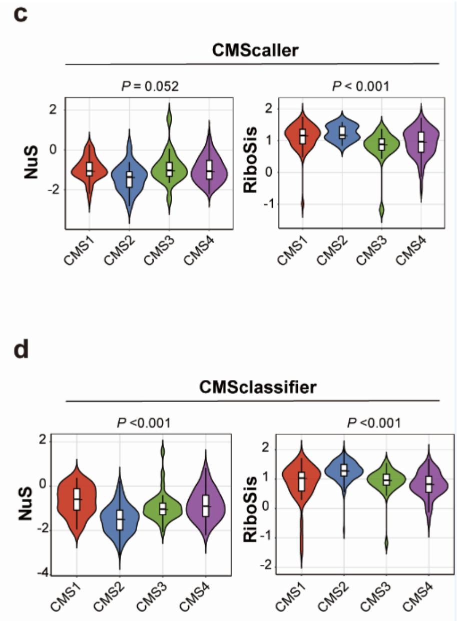

Supplementary Figure 11c-d

3. Association between TP53 alterations and NuS scores

Since p53 pathway activation is a core theme of the study, a formal statistical assessment of the relationship between TP53 status (mutations, deletions, or loss) and elevated NuS scores is warranted. Appropriate methods include Fisher's exact test, chi-squared test, or logistic regression (potentially adjusting for covariates). Such an analysis would clarify whether NuS primarily captures p53-dependent nucleolar stress responses in vivo or reflects more general defects in ribosome biogenesis that are independent of p53 status.

Our response:

We agree that a formal statistical assessment of the relationship between TP53 alterations and NuS is essential to clarify the extent to which NuS reflects p53-dependent versus broader nucleolar stress-associated processes.

We first examined the overlap between the nucleolar stress gene sets and the TP53 signaling gene set. Approximately 20% of nucleolar stress-associated genes overlapped with the TP53 pathway (Figure 2f), indicating that although NuS is associated with p53 signaling, it is not confined to this pathway and likely captures broader aspects of nucleolar biology. This observation further suggests that NuS reflects additional dimensions of nucleolar dysfunction beyond canonical p53 activation.

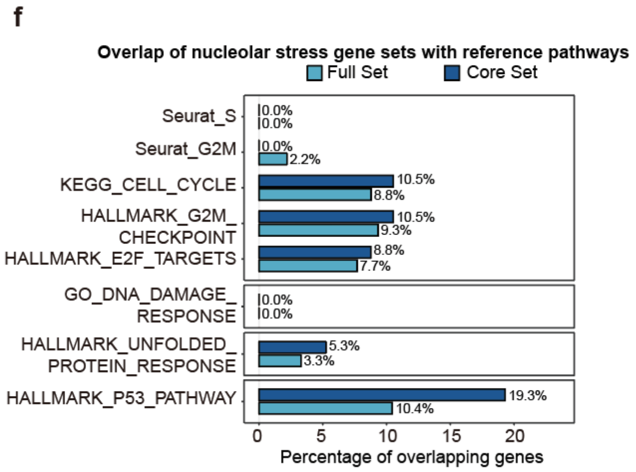

Figure 2f

We then performed additional analyses in TCGA cohorts to assess the association between TP53 status and NuS at the sample level. Tumors were stratified into wild-type (WT) and mutant (Mut) groups based on TP53 alteration status, and NuS distributions were compared using two-sided Wilcoxon rank-sum tests. As shown in Figure 6h and Supplementary Figure 11a-b, TP53-mutant tumors exhibited significantly lower NuS and higher RiboSis scores than TP53 wild-type tumors, consistent with attenuated nucleolar stress responses and enhanced ribosome biogenesis activity under conditions of impaired p53 function.

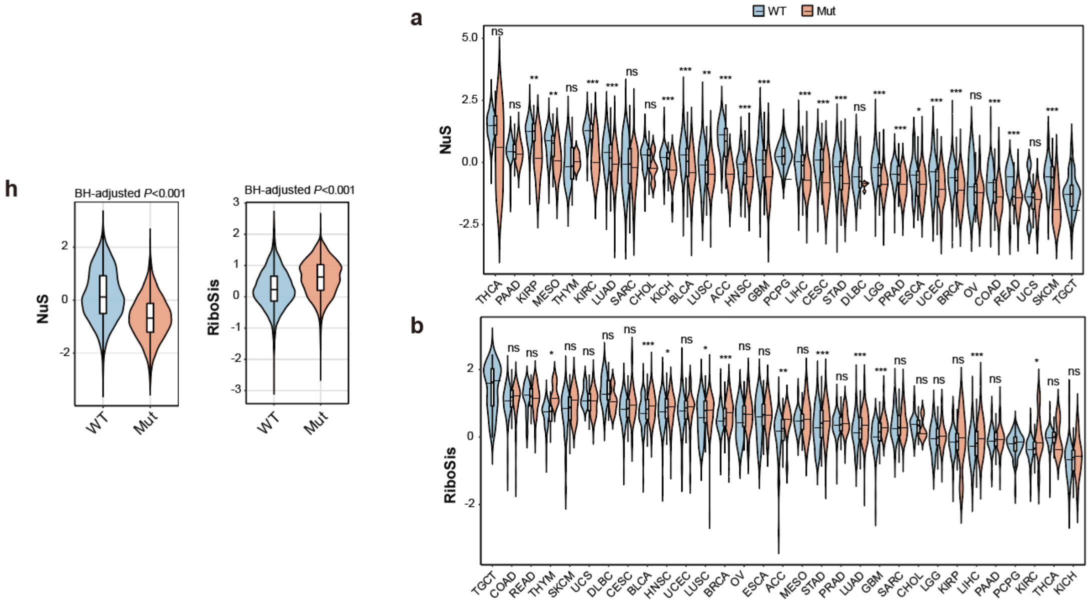

Figure 6h and Supplementary Figure 11a-b

To further determine whether NuS is merely a surrogate for p53 activity, we performed TP53-stratified analyses of the relationship between NuS and RiboSis. Notably, the inverse association between NuS and RiboSis was preserved in both TP53 wild-type and TP53-mutant

tumors, and remained particularly evident in the TP53-mutant subset (Supplementary Figure 11e-g). These findings indicate that NuS retains biological variability and functional relevance even in the absence of intact p53 signaling.

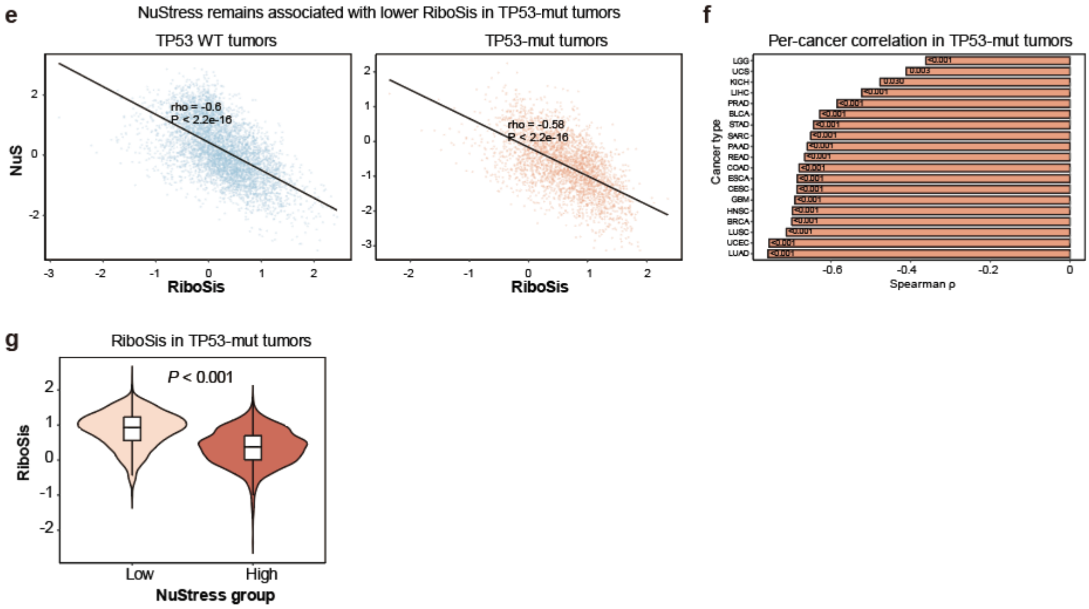

Supplementary Figure 11e-g

Together, these results demonstrate that although NuS is closely associated with TP53 status, it is not solely determined by p53 activity. Rather, NuS captures broader nucleolar functional states that extend beyond the canonical p53 pathway, including stress-associated and ribosome biogenesis-related processes.

4. Potential confounding by cell cycle heterogeneity

Ribosome biogenesis and rRNA processing genes are often cell cycle–regulated, with many downregulated during G0/G1 or mitosis. Consequently, NuS may partly reflect proliferation status or cell cycle phase distribution rather than specific nucleolar stress. This is particularly relevant in single-cell datasets, where cell cycle heterogeneity is prominent. To address this, the authors should compute established cell cycle scores (e.g., using Seurat's CellCycleScoring function with canonical S-phase and G2/M-phase gene sets) and evaluate correlations between NuS and cell cycle phase across datasets. If a strong covariance with proliferation is observed, this should be explicitly acknowledged. Ideally, the signature could be refined (e.g., by regressing out cell cycle effects) to better isolate nucleolar stress-specific signals. This control experiment would greatly bolster the biological specificity of NuS.

Our response:

We thank the reviewer for this important comment. We agree that cell-cycle heterogeneity is a

potential confounder, particularly in single-cell datasets, because ribosome biogenesis and rRNA-related programs are closely linked to proliferative state. At the same time, this relationship is also biologically relevant, as nucleolar activity is tightly coupled to cell-cycle progression, and cell-cycle-associated programs are among the major downstream processes altered during nucleolar stress. We therefore addressed this issue at both the gene-signature level and the single-cell scoring level.

First, we examined the overlap between NuS-associated genes and curated gene sets related to the cell cycle and other stress pathways. As shown in the revised manuscript (Figure 2f), fewer than 10% of NuS-associated genes overlapped with canonical cell-cycle genes, while overlap with DNA damage- and ER stress-related gene sets was even smaller. These findings argue that although NuS is related to pathways associated with proliferation and ribosome biogenesis, it is not simply a surrogate for cell-cycle activity or a generic stress-response signature.

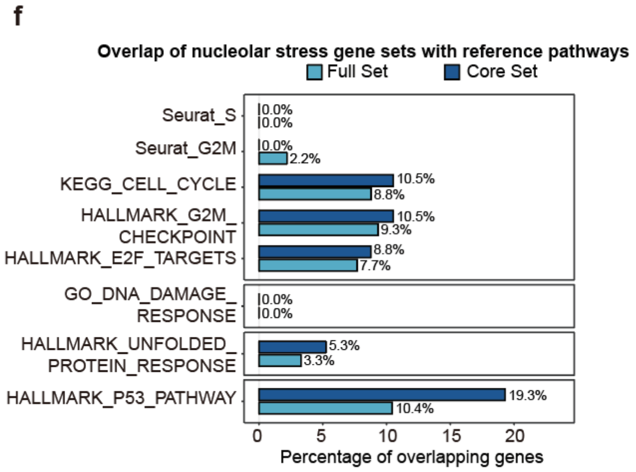

Figure 2f

Second, at the single-cell level, we directly evaluated the relationship between NuS and cell-cycle state in CRC epithelial cells. Using Seurat's CellCycleScoring function with canonical S-phase and G2/M-phase gene sets, we calculated cell-cycle scores and examined their association with NuS. We found that NuS was modestly negatively correlated with both S-phase and G2/M scores, and that cells assigned to S and G2/M phases exhibited lower NuS than cells in G1 phase (Figure 4k and Supplementary Figure 7e-g). These results indicate that NuS is influenced, at least in part, by cell-cycle state, and we now explicitly acknowledge this point in the revised manuscript. However, this effect did not fully account for the biological patterns we observed. Tumor-derived epithelial cells still showed lower NuS than matched normal epithelial cells within each cell-cycle phase (Figure 4l). Moreover, after regressing out the effects of S.Score and G2M.Score, substantial NuS-associated heterogeneity remained across epithelial cells (Figure 4m and Supplementary Figure 7h). Thus, although cell-cycle state contributes to

NuS variation, it does not fully explain the tumor-associated differences or the broader heterogeneity captured by the score.

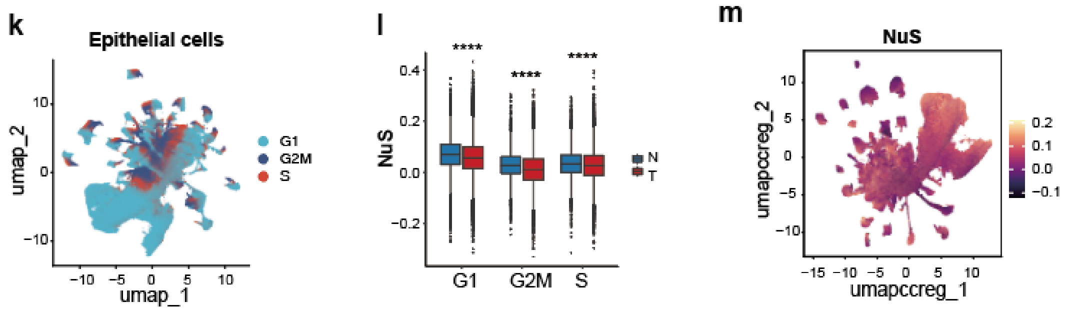

Figure 4k-m

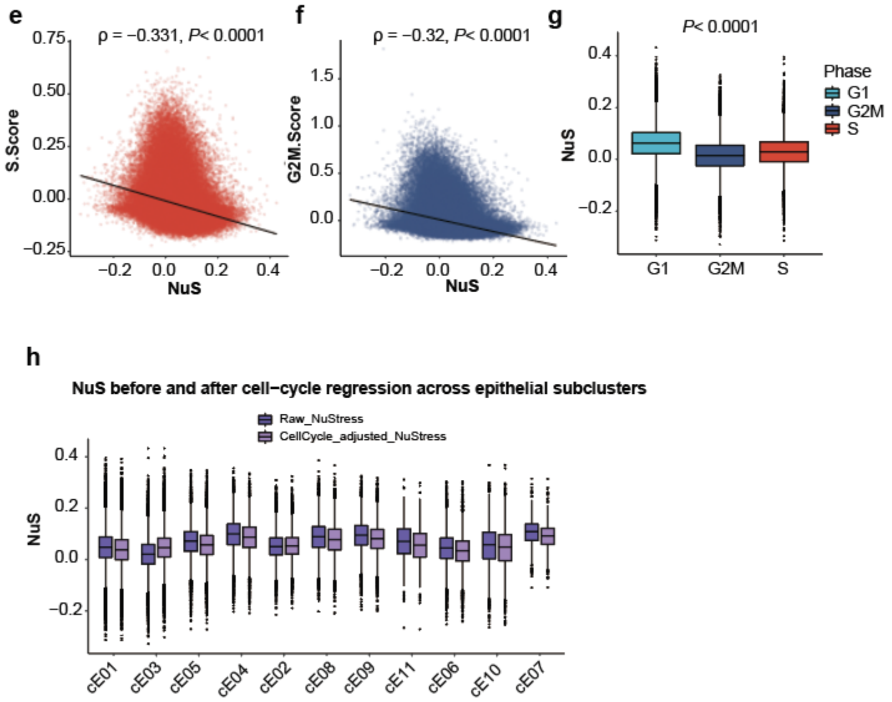

Supplementary Figure 7e-g

5. Distinguishing tumor-intrinsic effects from tissue-of-origin or microenvironmental influences

To confirm that differences in NuS (and related ribosome biogenesis signatures, RiboSis) reflect tumor-intrinsic oncogenic processes rather than baseline tissue-specific or microenvironmental nucleolar states, inclusion of a negative control cohort is recommended. Healthy tissues from multiple organs (e.g., from GTEx) would serve as an appropriate reference to benchmark NuS levels in non-malignant contexts. Without such controls, it remains uncertain whether observed elevations are truly cancer-specific. The study offers valuable insights into nucleolar stress in cancer, addressing these methodological and interpretative issues will markedly improve its

scientific impact, rigor, and potential for clinical translation.

Our response:

We thank the reviewer for this constructive comment. In the revised manuscript, we addressed this point by incorporating normal tissue controls from GTEx into our pan-cancer analyses. Specifically, to systematically characterize nucleolar function across human cancers, we computed NuS and RiboSis in treatment-naïve TCGA tumor cohorts together with normal tissues from GTEx, while excluding samples annotated as having received neoadjuvant therapy to minimize treatment-related confounding. This design provided a non-malignant reference across multiple tissue contexts.

Using this framework, we found that at the pan-cancer level, NuS was generally lower in tumors than in normal tissues, whereas RiboSis was broadly elevated in tumors (Figure 6b-c). Although baseline levels varied across tissue types, these patterns were not simply due to tissue-specific differences, but instead reflected tumor-intrinsic abnormalities in nucleolar functional state.

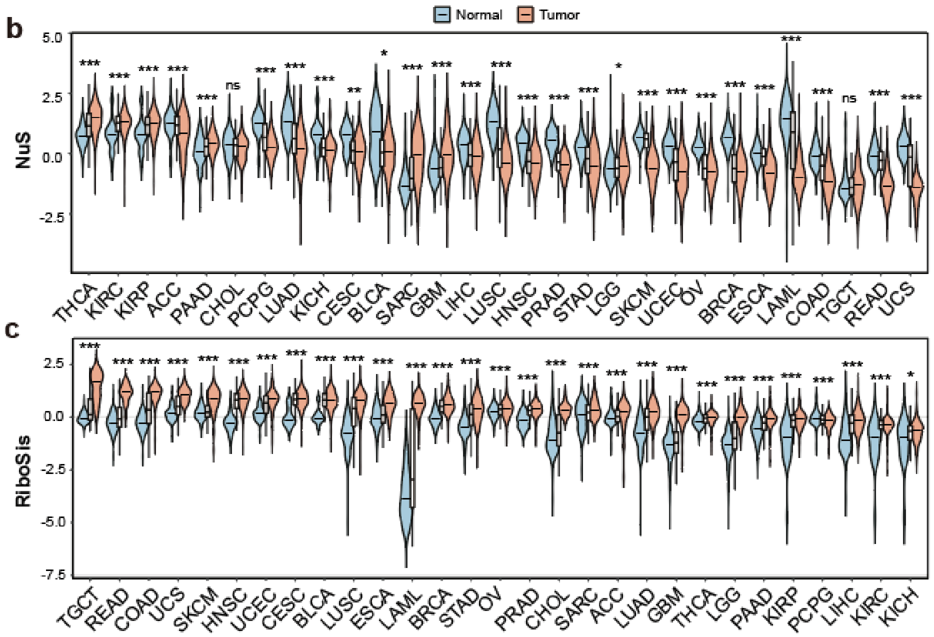

Figure 6b-c

In addition, our single-cell and spatial transcriptomic analyses further support a tumor-intrinsic component of these changes. In CRC single-cell data, tumor epithelial cells showed reduced NuS and increased RiboSis relative to matched normal epithelial cells, indicating that these differences are present within the malignant compartment itself rather than arising solely from tissue composition. Spatial transcriptomic analyses further showed that RiboSis was enriched in tumor regions, whereas NuS exhibited broader spatial heterogeneity. Together, these results suggest that although microenvironmental context contributes to nucleolar functional heterogeneity, the cancer-associated patterns we observed are not explained solely by tissue-of-origin or compositional differences.

Reviewer #2 (Remarks to the Author):

The authors describe a score for measuring nucleolar stress (NuS), applicable to a wide variety of data types. They evaluate the score in the context of colorectal cancer using both single-cell RNA-seq and spatial transcriptomic and further evaluate NuS using TCGA and CMap datasets, comparing with results given by the ribosomal scoring method RiboSis. Overall, I find the paper to be very well written, with robust analyses generated across numerous datasets and data types. I have the following specific comments, mainly around validation:

1. Lines 241 – 250 and Figures 4k-n. The authors stratify epithelial cell subsets where low-NuS cells show pathways related to ribosome biogenesis and rRNA metabolism that are downregulated. The claim is made that NuS is capturing functional variation not explained by Ribosome biogenesis alone. However, across the epithelial cell subsets, the RiboSis scores roughly seem to correspond to the NuS score. E.g. in the cE01 to cE03 subsets, both the RiboSis and NuS scores are elevated. Does the ribosome biogenesis etc. GO terms refer to the low RiboSis score here? What happens if the stratifications are performed based on RiboSis instead? What is the overlap of the cell stratifications, and do the GO terms look different?

Our response:

We thank the reviewer for this thoughtful comment. We agree that, in some epithelial subpopulations (cE02, cE04 and cE06), NuS and RiboSis show partially concordant patterns, and that this point should be clarified more explicitly.

To more accurately dissect the biological functions represented by epithelial cell subsets, we performed stratified analyses based on both NuS and RiboSis (Figure 4i-j and Figure 4n). Because of functional heterogeneity within epithelial cells, each subset may exhibit distinct combinations of nucleolar stress and ribosome biogenesis states, rather than conforming to a simple inverse relationship between the two scores. Accordingly, we did not interpret NuS and RiboSis as strictly opposing states, but rather as partially overlapping dimensions of nucleolar function.

To distinguish the biological programs represented by each score, we performed analyses while controlling for the other metric. Specifically, under comparable NuS or RiboSis backgrounds, we examined the pathways associated with high-RiboSis and high-NuS cells separately. These analyses showed that high-RiboSis cells were preferentially enriched for ribosome biogenesis- and rRNA metabolism-related pathways, whereas high-NuS cells were more strongly enriched for stress-associated programs, including responses to viral infection, external stimuli, and apoptotic signaling. Therefore, although both NuS and RiboSis may be elevated in certain epithelial cell subsets, they reflect overlapping but non-equivalent aspects of nucleolar function and highlight distinct features of epithelial cell state.

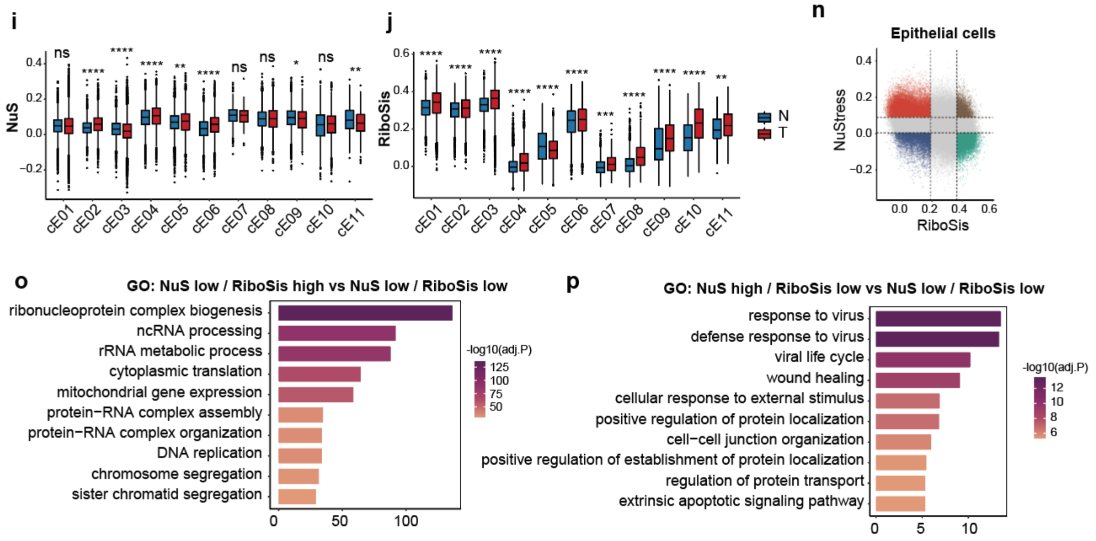

Figure 4i-j, n, o-p

We have revised the text to clarify that NuS does not represent a process fully independent of ribosome biogenesis, but rather a distinct and partially overlapping axis of nucleolar function. The key conclusion is therefore not that NuS and RiboSis are completely uncoupled, but that stratification based on these two scores identifies different functional enrichments and provides complementary biological information.

2. Lines 269-278: the therapy was associated with an increase in the NuS score. Do the authors know if the specific therapy is expected to induce nucleolar stress? That would help with validation. Similarly, the authors comment based on the RiboSis and NuS scores that the metastatic tumours may adopt a less proliferative state. This should be something that the authors could test with differential gene expression and GO term analysis?

Our response:

We thank the reviewer for this insightful comment. These suggestions help further strengthen the biological interpretation of our findings.

Regarding the therapy-associated increase in NuS, the neoadjuvant regimen used in this cohort was XELOX, which combines capecitabine and oxaliplatin. Our analyses, together with experimental validation, support oxaliplatin as a nucleolar stress-inducing agent. Specifically, we show that oxaliplatin suppresses rRNA transcription and activates nucleolar stress-associated responses in CRC models, with the magnitude of this response correlating with drug sensitivity (Figure 3 and Supplementary Figures 5-6). In addition, capecitabine is an oral prodrug of 5-FU. Consistent with previous reports that chemotherapeutic agents can disrupt ribosome biogenesis at multiple levels, we found that 5-FU also perturbs nucleolar function and increases NuS, although its primary effect is on pre-rRNA processing rather than direct

inhibition of rRNA transcription [1]. Together, these findings indicate that both components of the XELOX regimen can induce nucleolar stress, supporting the biological plausibility of the observed increase in NuS following treatment. We have clarified this point in the revised manuscript.

We also agree that the inference that metastatic tumors may adopt a less proliferative state should be supported more directly. To address this, we performed additional transcriptomic analyses comparing primary tumors and liver metastases. These analyses showed that metastatic lesions were characterized by reduced ribosome biogenesis-related programs together with enhanced metabolic remodeling (Supplementary Figure 8c), supporting the interpretation that metastatic tumors adopt a less proliferative, but potentially more adaptive, transcriptional state within the hepatic microenvironment.

References:

1. Burger K, Mühl B, Harasim T, Rohrmoser M, Malamoussi A, Orban M, Kellner M, Gruber-Eber A, Kremmer E, Hölzel M, Eick D. Chemotherapeutic drugs inhibit ribosome biogenesis at various levels. J Biol Chem. 2010 Apr 16;285(16):12416-25. doi: 10.1074/jbc.M109.074211. Epub 2010 Feb 16. PMID: 20159984; PMCID: PMC2852979.

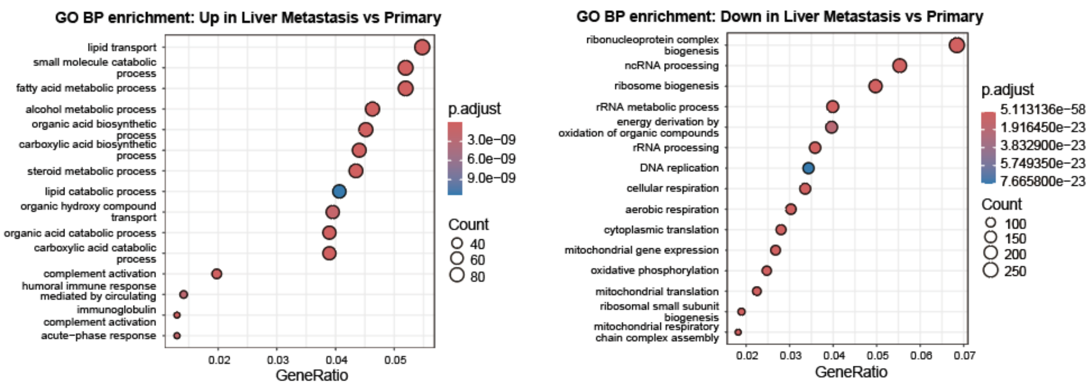

Supplementary Figure 8c

3. The authors evaluated the NuS score across drugs in CMap. In Potapova et al. eLife 2013 (PMID: 38099650), they scanned anti-cancer drugs for impact on nucleolar stress. As a validation, it would be good to compare the normality scores from Potapova et al. to the NuS scores, where there is overlap in the drugs tested.

Our response:

We thank the reviewer for this valuable suggestion. In response, we compared NuS scores with the morphological normality scores reported by Potapova et al. for the set of compounds shared between the two datasets. This analysis revealed an inverse correlation, such that higher NuS

scores were associated with lower normality scores, and this relationship was more pronounced at 10 μM than at 1 μM (Figure 7d) . Although the overall correlation was modest, the concordant trend supports agreement between transcriptome-based NuS scoring and morphology-based assessment of nucleolar perturbation.

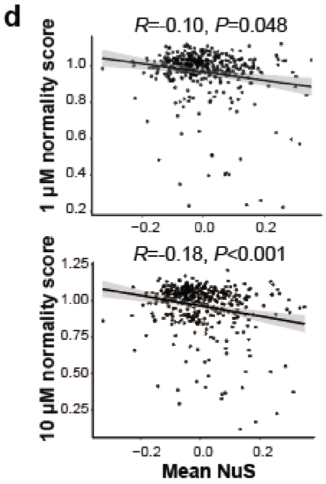

Figure 7d

In addition, our experimental validation showed that all selected compounds significantly reduced 47S pre-rRNA levels, even in cases where overt nucleolar structural changes were not readily detectable. This finding suggests that functional impairment of nucleolar activity can precede obvious morphological remodeling, and further supports NuS as a sensitive and complementary quantitative approach for assessing nucleolar stress.

4. The authors state in lines 336 – 337 that the recovery of Nutlin-3 is an internal validation, but I would like clarification here. In Goudarzi et al. it is stated "Nutlin-3 activates p53 by disruption of the p53-MDM2 protein-protein interaction and without inducing nucleolar stress". Can the authors clarify where they get their understanding of Nutlin-3 as a canonical nucleolar stress inducer? In either case, the comparison with the Potapova et al. dataset would make for a more robust testing of the NuS score.

Our response:

We thank the reviewer for this important comment. The statement in Goudarzi et al. is based on earlier studies showing that Nutlin-3 activates p53 through direct disruption of the p53–MDM2 interaction, rather than through upstream nucleolar perturbation. We therefore agree that Nutlin-3 should not be described as a canonical inducer of nucleolar stress. Instead, it is more appropriately viewed as a p53 activator that bypasses the canonical nucleolar stress-inducing step, as direct evidence that Nutlin-3 perturbs nucleolar function remains limited. Nevertheless, we consider Nutlin-3 to be a mechanistically informative comparator. Because

disruption of the MDM2-p53 axis represents a major downstream consequence of nucleolar stress signaling, Nutlin-3 provides a useful reference for distinguishing direct p53 activation from bona fide nucleolar stress. We have revised the manuscript accordingly to avoid overstating this point.

As suggested by the reviewer, we also compared NuS scores with the morphological normality scores for the overlapping compounds and observed an inverse correlation, with higher NuS associated with lower normality scores, particularly at 10 μM (Figure 7d). In addition, in the Potapova et al. dataset, Nutlin-3 and etoposide did not induce obvious nucleolar morphological changes after 24 h of treatment, which is consistent with our own SIM observations. This concordance suggests that some compounds may induce functional nucleolar perturbation without prominent morphological remodeling under these conditions, or that overt structural changes may require longer exposure.

5. In figure legends, the authors should indicate what statistical test is being used, for example, in Figure 3g, Figure 4k-l, Figure 5f, Figure 6a, Supp Figure 8c. etc.

Our response:

We thank the reviewer for this suggestion. In the revised manuscript, we have updated the relevant figure legends to explicitly indicate the statistical tests used for each analysis.

Reviewer #3 (Remarks to the Author):

I co-reviewed this manuscript with one of the reviewers who provided the listed reports. This is part of the Communications Biology initiative to facilitate training in peer review and to provide appropriate recognition for Early Career Researchers who co-review manuscripts.

Reviewer #4 (Remarks to the Author):

Chen el al. present a methodology and analytical framework to study nucleolar stress using a quantitative computational metric (NuS), followed by hypothesis-driven experimental testing and biological validation. The authors curate knowledge across the literature and multiple published expression datasets to identify a set of genes associated with nucleolar stress, addressing the current lack of a well-defined gene signature or ontology for this process. Conceptually, NuS is related to the RiboSis metric developed by another group; however, whereas RiboSis is derived from a predefined set of known ribosome biogenesis genes, this study seeks to define a new nucleolar stress–associated gene set and subsequently formalize it into a quantitative score. From my perspective, a key innovation of this work lies in its use of

computational analysis as a means for hypothesis generation and prioritization of downstream experimental validation.

My feedback:

1. Specificity of NuS to nucleolar stress

My main concern relates to the specificity of NuS for nucleolar stress, as opposed to reflecting more general cellular stress or damage responses. For example, in "Oxaliplatin induces nucleolar stress in colorectal cancer", NuS also increased following 5-FU treatment, despite the absence of clear nucleolar morphological changes. This raises the question of whether NuS selectively captures nucleolar stress or instead reflects broader transcriptional programs associated with cytotoxicity or stress responses.
The authors may wish to clarify this distinction, for example by (i) benchmarking NuS against established stress signatures (e.g. DNA damage, ER stress, or general p53 activation), or (ii) explicitly testing conditions known to induce non-nucleolar stress.

Our response:

We thank the reviewer for this important comment. We addressed this concern from two complementary angles.

First, in addition to its canonical effects on nucleotide metabolism, 5-FU has been reported to perturb RNA metabolism, particularly pre-rRNA processing and ribosome biogenesis [1]. Thus, the elevation of NuS in 5-FU-treated cells is biologically plausible even in the absence of overt nucleolar morphological changes at the 24 h time point examined. This observation suggests that certain drugs may induce nucleolar stress without immediate structural disruption, or may require longer exposure for overt nucleolar remodeling to become evident, while nucleolar function is already impaired. In this context, NuS may provide a sensitive assessmentt of functional nucleolar perturbation.

Second, we compared the NuS gene set with established signatures associated with DNA damage, ER stress, cell cycle, and p53 activation. Overlap with cell cycle, DNA damage, and ER stress signatures was limited, generally around 10% or less, arguing against NuS being a simple restatement of these broader stress-response pathways. By contrast, the greater overlap with p53 signaling is consistent with the well-established coupling between nucleolar stress and p53 activation (Figure 2f). We have clarified this point in the revised manuscript and now emphasize that NuS captures a transcriptional program associated with nucleolar dysfunction while remaining, as expected, partially connected to shared downstream stress signaling.

References:

1. Burger K, Mühl B, Harasim T, Rohrmoser M, Malamoussi A, Orban M, Kellner M, Gruber-Eber A, Kremmer E, Hölzel M, Eick D. Chemotherapeutic drugs inhibit ribosome biogenesis at various levels. J Biol Chem. 2010 Apr 16;285(16):12416-25. doi: 10.1074/jbc.M109.074211. Epub 2010 Feb 16. PMID: 20159984; PMCID: PMC2852979.

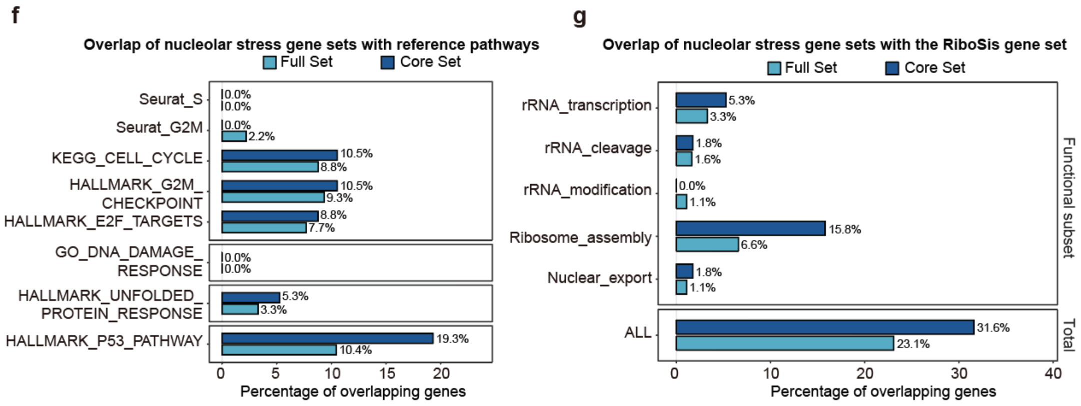

Figure 2f-g

2. p5 line 119 "18 upregulated and 33 downregulated" are not listed in Supplementary Data 2.

Our response:

Thank you for pointing this out. The high-confidence core gene set has now been listed in Supplementary Data 6. We have also revised the manuscript accordingly to ensure that these genes are clearly traceable in the supplementary materials.

3. p5 lines 123-126 I suggest restricting the DepMap essentiality analysis to the upregulated genes. Downregulation of a gene does not necessarily imply functional dependency or essentiality.

Our response:

We thank the reviewer for this valuable comment. We have revised the analysis to restrict DepMap essentiality assessment to the upregulated genes only. The corresponding results and text in the manuscript have been updated accordingly.

4. Relationship between NuS and RiboSis

While the manuscript appropriately positions NuS as complementary to RiboSis, it would benefit from a more explicit discussion of overlap and divergence between the two metrics. For example, quantifying gene-level overlap or correlation across datasets would help clarify how these scores capture distinct versus shared aspects of nucleolar and ribosome-related biology.

Our response:

We thank the reviewer for this constructive suggestion. In the revised manuscript, we have therefore expanded this section by explicitly quantifying their gene-level overlap and discussing their relationship in greater detail. Specifically, we found that approximately 30% of NuS-associated genes overlapped with the RiboSis gene set, indicating partial convergence but also substantial divergence between the two metrics (Figure 2g). In addition, across datasets, NuS and RiboSis showed only moderate correlation, further supporting that they capture related but non-identical aspects of nucleolar biology. Together, these results indicate that although both metrics are linked to nucleolar and ribosome-associated processes, NuS is not equivalent to RiboSis and instead captures broader features of nucleolar stress beyond ribosome biogenesis alone. The relevant results and discussion have been incorporated into the revised manuscript (Figure 2g, Figure 4 and Figure 6)

5. Causality versus association

The manuscript would benefit from clearer language regarding the relationship between NuS and nucleolar stress. In particular, it would be helpful to clarify whether changes in NuS are intended to precede, coincide with, or follow experimental indicators of nucleolar stress, and to explicitly state that NuS represents an associative metric rather than a causal measure.

Our response:

We thank the reviewer for this helpful suggestion. We agree that the relationship between NuS and nucleolar stress should be described more precisely. In the revised manuscript, we now explicitly state that NuS is an associative, expression-based metric that captures transcriptional remodeling associated with nucleolar stress, rather than a direct or causal measure of nucleolar stress itself. Accordingly, changes in NuS should be interpreted as evidence of a nucleolar stress-associated transcriptional state, but not as definitive proof that nucleolar stress has been induced in the absence of supporting functional or morphological assays. We have clarified this point in the Discussion.

We have also refined our description of the temporal relationship between NuS and experimental indicators of nucleolar stress. Because NuS is derived from gene expression changes, it most likely reflects concurrent and/or downstream transcriptional responses to nucleolar perturbation. Consistent with our experimental observations, including under 5-FU treatment, nucleolar functional impairment, such as reduced rRNA transcription or altered pre-rRNA processing, together with increased NuS, can occur in the absence of overt nucleolar morphological changes at early time points. These findings suggest that NuS may coincide with, or in some contexts follow, functional indicators of nucleolar stress, whereas morphological remodeling may emerge later or remain subtle depending on the perturbation.

RESPONSE TO REVIEWERS' COMMENTS

Reviewers' comments:

==========================

Reviewer #1 (Remarks to the Author):

After reviewing the authors' detailed response to my earlier remarks, I would like to express my satisfaction with the revisions implemented. The authors have addressed the raised concerns in a constructive and comprehensive manner, resulting in a noticeably improved manuscript that demonstrates greater rigor, analytical precision, and well-supported interpretations of the results and their broader implications.

The only remaining minor point pertains to Figure 5. To enhance visual consistency and enable more straightforward interpretation, I recommend aligning the color palettes assigned to the individual cell clusters. In particular, the palettes utilized in subpanels (b) and (e) should be standardized.

This minor adjustment would further elevate the clarity and professionalism of the presentation without altering the scientific content.

Our response:

We thank the reviewer for the positive assessment of our revised manuscript and for this helpful suggestion. We have standardized the color palettes used for the cell clusters in Figure 5b and Figure 5e to ensure visual consistency across the panels. This change is graphical and does not affect the underlying data, analyses, results, or conclusions.

Reviewer #2 (Remarks to the Author):

I thank the authors for addressing my comments.

Our response:

We thank the reviewer for their positive assessment.

Reviewer #3 (Remarks to the Author):

I co-reviewed this manuscript with one of the reviewers who provided the listed reports. This is part of the Communications Biology initiative to facilitate training in peer review and to provide appropriate recognition for Early Career Researchers who co-review manuscripts.

Our response:

We thank the co-reviewer for their contribution to the peer-review process and appreciate their careful evaluation of our manuscript.

Reviewer #4 (Remarks to the Author):

The authors have addressed all my comments.

Our response:

We thank the reviewer for their positive assessment.
